# Complete genome sequence of *Pasteurella multocida* subsp*. septica* isolated from a sepsis patient in Thailand

**DOI:** 10.1128/mra.00523-26

**Published:** 2026-08-28

**Authors:** Supawee Kinnonkok, Kiatthida Tinthalang, Jenjira Rujidamp, Supphachoke Khemla, Barameht Piralam, Orapan Sripichai, Pisut Pongchaikul

**Affiliations:** 1 Department of Medical Sciences, Ministry of Public Health, National Institute of Health (NIH), Nonthaburi, Thailand; 2 Division of Infectious Diseases, Department of Internal Medicine, Nakhon Phanom Hospital, Nakhon Phanom, Nakhon Phanom Province, Thailand; 3 Nakhon Phanom Provincial Health Office, Nakhon Phanom, Thailand; 4 Chakri Naruebodindra Medical Institute, Faculty of Medicine, Ramathibodi Hospital, Mahidol University 26685, Samut Prakarn, Thailand; 5 Integrative Computational BioScience Center, Mahidol University 26685, Nakhon Pathom, Thailand; 6 Institute of Infection, Veterinary and Ecological Sciences, University of Liverpool 4591, Liverpool, United Kingdom; University of Pittsburgh School of Medicine, Pittsburgh, Pennsylvania, USA

**Keywords:** sepsis, *Pasteurella*

## Abstract

*Pasteurella multocida* is a rare cause of human infection most often associated with animal bites or scratches. We report the complete genome sequence of *Pasteurella multocida* subsp. *septica* strain 0092 recovered from the blood culture of a sepsis patient in Nakhon Phanom, Thailand in 2022. The genome totals 2,392,800 bp.

## ANNOUNCEMENT

*Pasteurella multocida* is an anaerobic gram-negative coccobacillus known as oral commensal in cats and dogs. *P. multocida* can be divided into five capsular serotypes (A, B, D, E, and F) (1). Up until now, over 2,500 genomes are publicly available; however, approximately 10% of the genome was from human. Among human-associated isolates, subspecies *septica* has been reported as one of the most prevalent (2).

Strain 0092 was isolated from the blood culture drawn from a patient with sepsis at Nakhon Phanom Hospital, Thailand in 2022. The primary blood culture isolate was recovered on 5% sheep blood agar at 35 ± 2°C in ambient air for 18 h and identified to the species level by the BD Phoenix Automated Identification System (BD Diagnostic Systems, Sparks, MD).

For the isolate preparation, colonies were revived from −80°C frozen stock in rich media with 15% glycerol and sub-cultured on a blood agar plate. The plate was incubated at 35 ± 2°C in ambient air for 20–24 h to ensure colony purity and viability prior to subsequent steps. Antimicrobial susceptibility testing interpreted according to Clinical and Laboratory Standards Institute (CLSI) M45 (3rd edition) and European Committee on Antimicrobial Susceptibility Testing (EUCAST) (version 16) showed susceptibility to co-trimoxazole and ceftriaxone; otherwise, it showed non-susceptible phenotypes.

Genomic DNA was extracted from approximately 1 × 10⁹ bacterial cells, adjusted to McFarland standard 3.5–4.0, and suspended in 1 mL of phosphate-buffered saline (PBS) using the ZymoBIOMICS DNA Miniprep Kit (Zymo Research, Irvine, CA, USA) following the manufacturer’s instructions. Libraries were prepared with the Rapid Barcoding Kit SQK-RBK004 (Oxford Nanopore Technologies). Because SQK-RBK004 is a rapid, transposase-based kit (not a ligation kit), the genomic DNA was neither mechanically sheared nor size-selected prior to library preparation. The library was sequenced on a FLO-MIN106 V9.4.1 flow cell using a MinION Mk1C (Oxford Nanopore Technology). Base-calling was performed in MinKNOW software using high-accuracy model. Sequencing produced 126,562 reads, with a read *N*50 of 10,425 bp, corresponding to approximately 300× coverage.

*De novo* assembly was generated with Flye v2.9.5-b1801 (3). Genome contamination and the completeness were assessed with CheckM2 version 1.1.0 (4) and BUSCO version 6.1.0 (5), respectively. Annotation was performed with Bakta version 1.9.4 (6). Default parameters were used unless otherwise specified. Taxonomic assignment was confirmed by average nucleotide identity (ANI). The strain 0092 shared 98.73% ANI with *P. multocida* subsp. *septica* calculated with EZBioCloud above 96% threshold.

The draft genome consists of a circular chromosome and a circular plasmid with a total length of 2,351,053 bp and 41,747. The guanine-cytosine (GC) content was 40.3% (chromosome) and 32.9% (plasmid) (Table 1).

**TABLE 1:** Genome characteristics of *P. multocida* subsp. *septica* strain 0092

Strain	Strain 0092	
ANI hit	*Pasteurella multocida* subsp. *septica*	
	Chromosome	Plasmid
GC content (%)	40.3	32.9
Size (bp)	2,351,053	41,747
tRNAs	58	0
rRNAs	19	0
CDSs	3,436	71
CRISPR region	3	0

Screening for antibiotic resistance determinants using CARD (7), AMRprofiler (8), and AMRfinder (9),identified a cAMP-activated global transcriptional regulator, with 98% sequence coverage and 86% identity. In *Escherichia coli*, this regulator modulates expression of *mdtEF* efflux pump (10). Its presence may contribute to the reduced susceptibility observed for several antimicrobial agents tested.

Strain 0092 expands the small set of human-derived *P. multocida* subsp. *septica* genomes from mainland Southeast Asia and provides a regional reference for genomic surveillance of invasive pasteurellosis.

## Data Availability

The draft genome for *P. multocida* subsp. *septica* strain 0092 has been deposited to NCBI under BioProject accession number PRJNA1444785 (BioSample accession number SAMN56769409; Sequence Read Archive accession number SRR39247279).

## References

[1] Carter GR. 1955. Studies on *Pasteurella multocida*. I. A hemagglutination test for the identification of serological types. Am J Vet Res 16:481–484. 13238757

[2] Smallman TR, Perlaza-Jiménez L, Wang X, Korman TM, Kotsanas D, Gibson JS, Turni C, Harper M, Boyce JD. 2024. Pathogenomic analysis and characterization of *Pasteurella multocida* strains recovered from human infections. Microbiol Spectr 12:e03805-23. doi:10.1128/spectrum.03805-23 PMC10986470 38426766

[3] Freire B, Ladra S, Parama JR. 2022. Memory-efficient assembly using flye. IEEE/ACM Trans Comput Biol Bioinform 19:3564–3577. doi:10.1109/TCBB.2021.3108843 34469305

[4] Chklovski A, Parks DH, Woodcroft BJ, Tyson GW. 2023. CheckM2: a rapid, scalable and accurate tool for assessing microbial genome quality using machine learning. Nat Methods 20:1203–1212. doi:10.1038/s41592-023-01940-w 37500759

[5] Webster J, Chapman TA. 2026. Buscogeny: a BUSCO leveraged phylogenomic tree builder. Int Microbiol 29:255–267. doi:10.1007/s10123-025-00752-6 PMC12904889 41575681

[6] Schwengers O, Jelonek L, Dieckmann MA, Beyvers S, Blom J, Goesmann A. 2021. Bakta: rapid and standardized annotation of bacterial genomes via alignment-free sequence identification. Microb Genom 7:000685. doi:10.1099/mgen.0.000685 PMC8743544 34739369

[7] Alcock BP, Huynh W, Chalil R, Smith KW, Raphenya AR, Wlodarski MA, Edalatmand A, Petkau A, Syed SA, Tsang KK, et al.. 2023. CARD 2023: expanded curation, support for machine learning, and resistome prediction at the Comprehensive Antibiotic Resistance Database. Nucleic Acids Res 51:D690–D699. doi:10.1093/nar/gkac920 PMC9825576 36263822

[8] Skoulakis A, Kostoula V, Daniilidis K, Gkountinoudis CG, Petinaki E, Hatzigeorgiou AG. 2025. AmrProfiler: a comprehensive tool for identifying antimicrobial resistance genes and mutations across species. Nucleic Acids Res 53:W20–W31. doi:10.1093/nar/gkaf400 PMC12230725 40347103

[9] Feldgarden M, Brover V, Haft DH, Prasad AB, Slotta DJ, Tolstoy I, Tyson GH, Zhao S, Hsu CH, McDermott PF, Tadesse DA, Morales C, Simmons M, Tillman G, Wasilenko J, Folster JP, Klimke W. 2019. Validating the AMRFinder tool and resistance gene database by using antimicrobial resistance genotype-phenotype correlations in a collection of isolates. Antimicrob Agents Chemother 63:e00483-19. doi:10.1128/AAC.00483-19 PMC6811410 31427293

[10] Nishino K, Senda Y, Yamaguchi A. 2008. CRP regulator modulates multidrug resistance of *Escherichia coli* by repressing the mdtEF multidrug efflux genes. J Antibiot (Tokyo) 61:120–127. doi:10.1038/ja.2008.120 18503189

